# Marker-assisted pyramiding of two major, broad-spectrum bacterial blight resistance genes, *Xa21* and *Xa33* into an elite maintainer line of rice, DRR17B

**DOI:** 10.1371/journal.pone.0201271

**Published:** 2018-10-25

**Authors:** Balachiranjeevi C. H., Bhaskar Naik S., Abhilash Kumar V., Harika G., Mahadev Swamy H. K., Hajira Sk, Dilip Kumar T., Anila M., Kale R. R., Yugender A., Pranathi K., Koushik M. B. V. N., Suneetha K., Bhadana V. P., Hariprasad A. S., Laha G. S., Rekha G., Balachandran S. M., Madhav M. S., Senguttuvel P., Fiyaz A. R., Viraktamath B. C., Giri A., Swamy B. P. M., Jauhar Ali, Sundaram R. M.

**Affiliations:** 1 International Rice Research Institute, Metro Manila, Philippines; 2 Biotechnology, Crop Improvement, Indian Institute of Rice Research, Indian Council of Agriculture Research, Hyderabad, India; 3 Department of Plant Breeding, International Rice Research Institute-South Asia Hub, ICRISAT, Patancheru, India; 4 Division of Crop Improvement, ICAR-Sugarcane Breeding Institute, Coimbatore, Tamil Nadu, India; 5 ICAR-Indian Institute of Agricultural Biotechnology, PDU Campus, IINRG, Namkum, Ranchi, Jharkhand, India; 6 Centre for Biotechnology, Institute of PG Studies and Research, Jawaharlal Nehru Technological University, Mahaveer Marg, Hyderabad, India; Louisiana State University, UNITED STATES

## Abstract

Bacterial blight (BB) disease reduces the yield of rice varieties and hybrids considerably in many tropical rice growing countries like India. The present study highlights the development of durable BB resistance into the background of an elite maintainer of rice, DRR17B, by incorporating two major dominant genes, *Xa21* and *Xa33* through marker-assisted backcross breeding (MABB). Through two sets of backcrosses, the two BB resistance genes were transferred separately to DRR17B. In this process, at each stage of backcrossing, foreground selection was carried out for the target resistance genes and for non-fertility restorer alleles concerning the major fertility restorer genes *Rf3* and *Rf4*, using gene-specific PCR-based markers, while background selection was done using a set of 61 and 64 parental polymorphic SSR markers respectively. Backcross derived lines possessing either *Xa21* or *Xa33* along with maximum genome recovery of DRR17B were identified at BC_3_F_1_ generation and selfed to develop BC_3_F_2_s. Plants harboring *Xa21* or *Xa33* in homozygous condition were identified among BC_3_F_2_s and were intercrossed with each other to combine both the genes. The intercross F_1_ plants (ICF_1_) were selfed and the intercross F_2_(ICF_2_) plants possessing both *Xa21* and *Xa33* in homozygous condition were identified with the help of markers. They were then advanced further by selfing until ICF_4_ generation. Selected ICF_4_ lines were evaluated for their resistance against BB with eight virulent isolates and for key agro-morphological traits. Six promising two-gene pyramiding lines of DRR17B with high level of BB resistance and agro-morphological attributes similar or superior to DRR17B with complete maintenance ability have been identified. These lines with elevated level of durable resistance may be handy tool for BB resistance breeding.

## Introduction

Rice production needed to be increased 42% by 2050to feed the demands of an ever-increasing human population globally [1, 2]. Exploitation of heterosis for grain yield through hybrid rice technology is one of the feasible options to enhance rice production and rice hybrids have15-20%yield advantage over inbreeds [3]. Even though rice hybrids were introduced in India in the early 1990s, their adoption has been slow and presently hybrid rice is cultivated in a limited area of 2.5 million ha. One of the principal reasons for slow adoption of rice hybrids in India is their susceptibility to major rice diseases like bacterial blight (BB) and blast [4]. Most of the commercial rice hybrids that have been released and cultivated in India do not possess broad spectrum resistance for BB disease [5].

BB disease is caused by a gram-negative bacterium called *Xanthomonas oryzae* pv. *Oryzae* (*Xoo*). It is one of the most devastating diseases in rice [6]. The bacterium infects rice at maximum tillering stage, leading to water soaking lesions (blighting) on the leaves, which gradually enlarge, wilts and causes yield losses ranging from 74 to 81% [7]. Application of chemicals or antibiotics against is very costly and is not very effective [8, 9]. Breeding BB resistant rice varieties and hybrids is the best strategy for managing the BB disease in rice [10]. To date, at least 41 BB resistance genes have been identified,and some of them *viz*., *Xa4*, *xa5*, *xa13*, *Xa21* have been extensively used for development of BB resistant rice varieties [11, 12, 13, 14, 15] (**Table 1**). However, large scale and long-term cultivation of varieties and hybrids with a single gene results in the breakdown of resistance due to a high degree of pathogenic variation [12, 16, 17]. Pyramiding of two or three *Xa* genes can enhance the durability and spectrum of resistance against BB [18, 19].

**Table 1:** Agroclimatic zones according ICAR- IIRR [4] and ICAR-NARP [20], existing pathotypes and recommended genes.

S.No.	Agro climatic Rice growing zones (AZ) ICAR-IIRR (ACRIP)	Agro climatic zone (AZ) ICAR-NARP	State	Number of *Xoo* Pathotypes	Recommended Resistance genes and gene combinations
1	Zone I	AZ6-AZ9	Himachal Pradesh	**2** (1 & 6)	*xa13*, *Xa21*, *xa5+xa13*, *xa13+Xa21*, *Xa4+xa5+xa13*, *Xa4+xa5+Xa21*, *xa5+xa13+Xa21 & Xa4+xa5+xa13+Xa21*
2	Zone II	AZ10-AZ14	Punjab	**15** (1,2,5,6,7 8,9, 11, 12,14,17,19 20,21 & 22)	*xa5+xa13+Xa21 & Xa4+xa5+xa13+Xa21*
3	Zone II	AZ15-AZ16	Haryana	**9** (1,2,5,14,17,18,19,21 &22)	*xa5+xa13+Xa21 & Xa4+xa5+xa13+Xa21*
4	Zone I, Zone II, Zone III	AZ26-AZ35	Uttaranchal & Uttar Pradesh	**12**(2,3,6,7,10,11,12,14,17,19,20 &22)	*xa5+xa13+Xa21 & Xa4+xa5+xa13+Xa21*
5	Zone I, Zone III	AZ36-AZ41	West Bengal	**8** (1,4,6,7,12,17,19 &22)	*xa5+xa13+Xa21 & Xa4+xa5+xa13+Xa21*
6	Zone IV	AZ42-AZ47	Assam	8 (4,7,11,14,17,19,21 & 22)	*xa5+xa13+Xa21 & Xa4+xa5+xa13+Xa21*
7	Zone IV	AZ53	Tripura	**11** (1,2,7,9,11,13,14,17,19,21 & 22)	*xa5+xa13+Xa21 & Xa4+xa5+xa13+Xa21*
8	Zone III	AZ54-AZ59	Bihar & Jharkhand	2 (19 & 22)	*xa5+xa13+Xa21 & Xa4+xa5+xa13+Xa21*
9	Zone III	AZ60-AZ69	Odisha	**8** (1,2,4,7,11,16,17 & 19)	*Xa4+xa5+xa13*, *Xa4+xa5+Xa21*, *xa5+xa13+Xa21 & Xa4+xa5+xa13+Xa21*
10	Zone V	AZ 70-AZ81	Madhaya Pradesh & Chattisgarh	**7** (1,2,6,14,17,19 & 22)	*xa5+xa13+Xa21 & Xa4+xa5+xa13+Xa21*
11	Zone VI	AZ82-AZ89	Gujarat	**6** (2,3,6,7,17 & 19)	*Xa4+xa5+xa13*, *Xa4+xa5+Xa21*, *xa5+xa13+Xa21 & Xa4+xa5+xa13+Xa21*
12	Zone V and VI	AZ90-AZ98	Maharashtra	**5** (6,14,17,19 & 22)	*xa5+xa13+Xa21 & Xa4+xa5+xa13+Xa21*
13	Zone I, Zone VII	AZ99-AZ108	Karnataka	**4** (2,5,19 & 22)	*xa5+xa13+Xa21 & Xa4+xa5+xa13+Xa21*
14	Zone VII	AZ109-AZ113	Kerala	**9** (1,2,6,7,12,14,17,19 & 22)	*xa5+xa13+Xa21 & Xa4+xa5+xa13+Xa21*
15	Zone I, Zone VII	AZ114-AZ120	Andhra Pradesh & Telangana	**12** (1,5,6,7,9,11,12,14,17,19,21 &22)	*xa5+xa13+Xa21 & Xa4+xa5+xa13+Xa21*
16	Zone I, Zone VII	AZ121-AZ127	Tamil Nadu	**11** (1,5,6,7,9,11,12,14,17,19 & 21)	*xa5+xa13+Xa21 & Xa4+xa5+xa13+Xa21*

Zone I: Hilly Areas, Zone II: Northern, Zone III: Eastern, Zone IV: North Eastern, Zone V: Central, Zone VI: Western, Zone VII: Southern

The major BB resistance gene, ‘*Xa21’* was identified from *Oryza longistaminata*. It is located on chromosome 11 and a tightly linked to gene-specific marker pTA248 [21]. Similarly, ‘*Xa33’* was identified from *Oryza nivara*. It is located on chromosome 7 and tightly linked to a marker RMWR7.6 [22]. These markers can be used in marker-assisted breeding to introgress *Xa21* and *Xa33* genes into different rice varieties and hybrid parental lines. These two genes are found to be highly effective against several isolates of *Xoo* from India and hence, are ideal choices for pyramiding into popular rice varieties or hybrids through marker-assisted breeding.

DRR17B is a fine grain type and medium duration, stable promising maintainer line developed by ICAR-Indian Institute of Rice Research, Hyderabad, India [23]. It is however highly susceptible to BB of rice. In the present study, two major dominant BB resistance genes, *Xa21* and *Xa33* were introgressed into the genetic background of DRR17B through marker-assisted backcross breeding to develop improved DRR17B lines with broad spectrum resistance against BB.

## Materials and methods

### Plant materials

‘Improved Samba Mahsuri’ (**ISM**) is a recently released high-yielding and fine grain rice variety possessing BB genes, *xa5*, *xa13*, *and Xa21* [18]. It was used as a donor for *Xa21* [23]. A Near Isogenic Line (NIL) of ‘Samba Mahsuri’ (FBR1-15EM) served as the donor for *Xa33* [22]. The popular but BB susceptible maintainer line DRR17B (APMS6B/BPT5204/IR69628B) was used as the recurrent parent. It was developed by ICAR-Indian Institute of Rice Research (IIRR), Hyderabad (17.3200° N, 78.3939° E), India.

### Strategy for marker-assisted introgression of *Xa21* and *Xa33* into DRR17B

**Fig 1:**
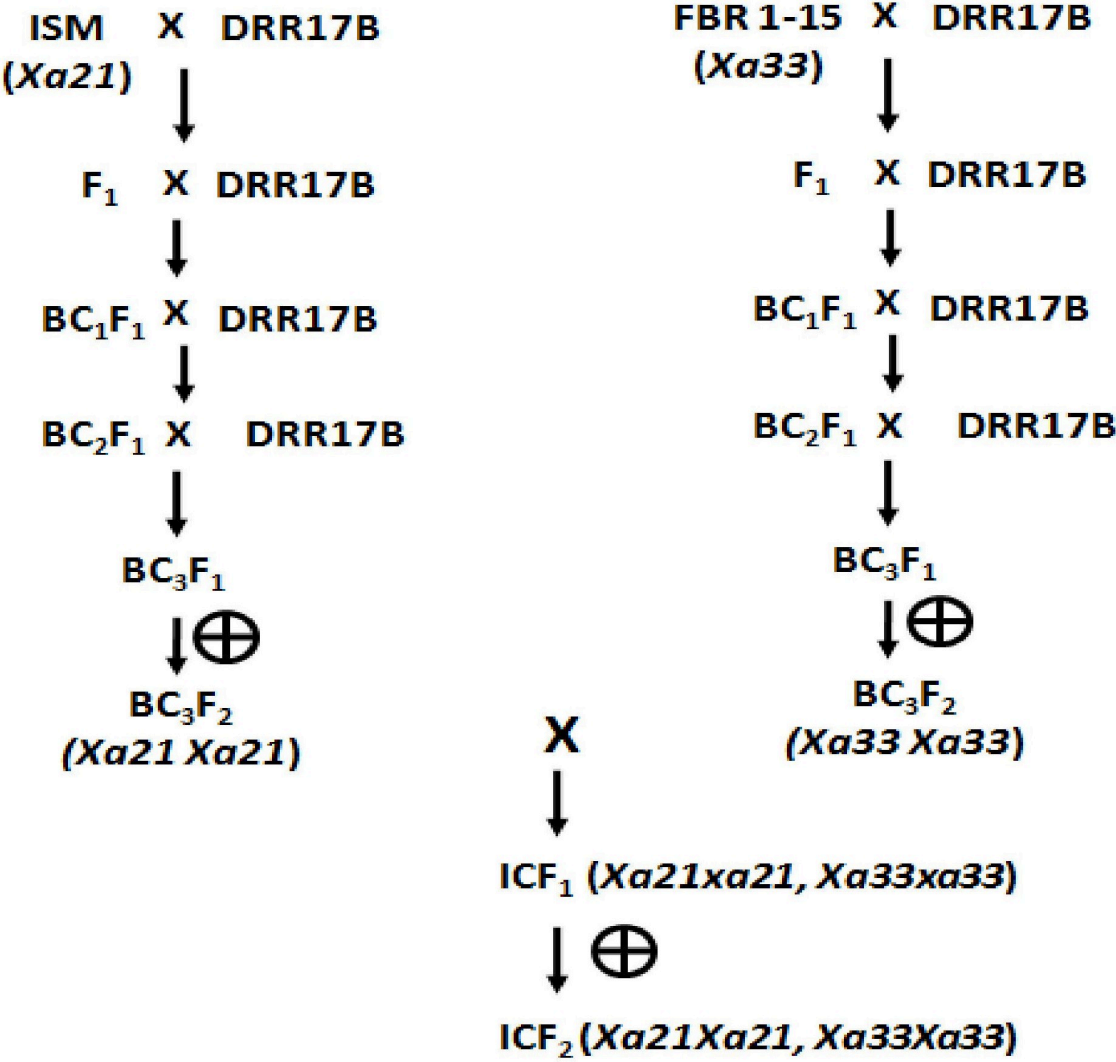
Marker-assisted backcrossing scheme adopted in the study.

Marker-assisted backcross breeding strategy was adapted for targeted introgression of *Xa21* and *Xa33* genes into the genetic background of the elite maintainer line of rice, DRR17B. Each of these genes was separately introgressed into DRR17B through two sets of crosses, i.e., Cross I, *viz*., DRR17B/**ISM** and Cross II, *viz*., DRR17B/FBR1-15 (**Fig 1**). The F_1_s obtained from the two crosses were analysed by extracting DNA through the method described by [24] and using that DNA by keeping Polymerase Chain reaction with gene-specific markers pTA248 (specific for *Xa21*; [21]) and RMWR7.6 (specific for *Xa33*; [22]) to identify ‘true’ heterozygotes. The ‘true’ F_1_s were backcrossed with the recurrent parent DRR17B to generate BC_1_F_1_s, which were then screened for the presence of the target resistance genes using the gene-specific markers. The positive plants for *Xa21* and *Xa33* were selected and further screened for the non-presence of major fertility restorer genes, *Rf4* and *Rf3* using tightly linked markers, viz., DRCG-*RF4*-14 and DRRM-*RF3*-10, respectively [25]. BC_1_F_1_ plants possessing BB genes and a non-restoring allele concerning *Rf4* and *Rf3* in homozygous condition were selected following the procedure described by [23]. These plants were later screened with a set of parental polymorphic SSR markers (61 markers specific to the cross DRR17B/**ISM** and 64 markers specific for the cross DRR17B/FBR1-15EM) through background selection to identify a single BC_1_F_1_ plant from each cross possessing maximum recovery of the recurrent parent genome. The selected plant was backcrossed once again with DRR17B.

The process of marker-assisted backcrossing was repeated until BC_3_ generation, and BC_3_F_1_ plants of DRR17B possessing either *Xa21* or *Xa33* and maximum recovery of recurrent parent genome were then selfed to obtain BC_3_F_2_s. Plants homozygous for either *Xa21* or *Xa33* were identified among the BC_3_F_2_ plants and the best plants from the two crosses were intercrossed to obtain intercross F_1_s (i.e., ICF_1_s). ‘True’ ICF_1_ plants were identified by screening with molecular markers specific for *Xa21* and *Xa33* and were then selfed to generate intercross F_2_s (i.e., ICF_2_s). Plants homozygous for both *Xa21* and *Xa33* were identified among the ICF_2_ plants using the gene-specific markers. The identified plants were advanced through the pedigree method of selection (involving selfing followed by morphological trait-based visual selection) up to ICF_4_generation. Marker-assisted selection procedures were followed as recommended by [21] and [22] for detection of *Xa21* and *Xa33* genes, while the protocol described by [23] was adopted for background selection and detection of non-restoring alleles of *Rf4* and *Rf3*.

### Screening for BB resistance

Eight virulent isolates of the BB pathogen, *Xanthomonas oryzae* pv. *oryzae* (*Xoo*) collected from BB disease endemic across major rice cultivation agro climatic locations in India, *viz*., IX-020 (Hyderabad, Telangana), IX-133 (Raipur, Chhattisgarh), IX-027 (Chinsurah, West Bengal), IX-200 (Pantnagar, Uttarakhand), IX-002 (Faizabad, Uttar Pradesh), IX-403 (Thanjavur, Tamil Nadu), IX-090 (Ludhiana, Punjab) and IX-281 (Tanuku, Andhra Pradesh) were used to screen the ICF_4_ lines of DRR17B (possessing the gene combinations *Xa21*+*Xa33*, *Xa21* alone or *Xa33* alone) along with the donor parents/resistant check, ‘**ISM**’ (possessing *xa5*+*xa13*+*Xa21*), FBR1-15 (possessing *Xa33*) and BB recurrent parent and susceptible check (DRR17B and TN1) were screened under glasshouse conditions for their resistance/susceptibility against BB. The *Xoo* strains were cultured and stored as described by [16]. The rice plants were clip-inoculated with a bacterial suspension of 10^8−9^ CFU/ml at maximum tillering stage (45 to 55 days after transplanting) through the methodology of [26]. Approximately, 5 to 10 leaves were inoculated per plant, and the disease reaction was scored 14 days after inoculation [27]. In addition to measurement of BB lesion length, the disease score was calculated as per IRRI Standard Evaluation System (SES) scale, which is based on percent diseased leaf area [28].

### Screening for agro-morphological traits

Improved lines (ILs) of DRR17B (ICF_4_) were field evaluated in randomized complete block design in Kharif 2014 (i.e. July-October/Wet season 2014) for the following agro-morphological traits involving days to 50% flowering (days), plant height (cm), number of productive tillers (No.), panicle length (cm), grains per panicle (No.) and spikelet fertility. Each entry was planted in 20 rows of 4m length with a spacing of 15 x 20 cm between rows and within rows. Days to 50 percent flowering was recorded based on number of days from sowing to 50% population flowering on a whole plot basis. Plant height (cm), number of productive tillers (No.) and panicle length (cm) were recorded from 5 competitive plants from each plot chosen at random and the mean values computed for different lines. Five individual panicles harvested separately from five plants were collected to compute for the average grain number per panicle (No.). The ILs were crossed with IR58025A line and evaluated for spikelet fertility based on seed setting of each cross. The percentage was calculated based on seed setting per panicle as described in [23].

### Statistical analysis

Agro-morphological and phenotypic BB screening data were analysed using standard procedures by calculating Mean, significant standard error of Mean (S.E.M ±), Analysis of variance (ANOVA) and Least Significance Difference (LSD) [29]. Analysis of variance (ANOVA) and Duncan’s multiple range test (DMRT) and Least Significance Difference (LSD) at 5% level of significance, significant standard error of Mean (S.E.M ±) were calculated by using MS Excel and Statistical computer software Statistix8.1 [30] software to analyze the variation between ILs and parents.

## Results

### Marker-assisted transfer of *Xa21* and *Xa33* into DRR17B

**Fig 2:**
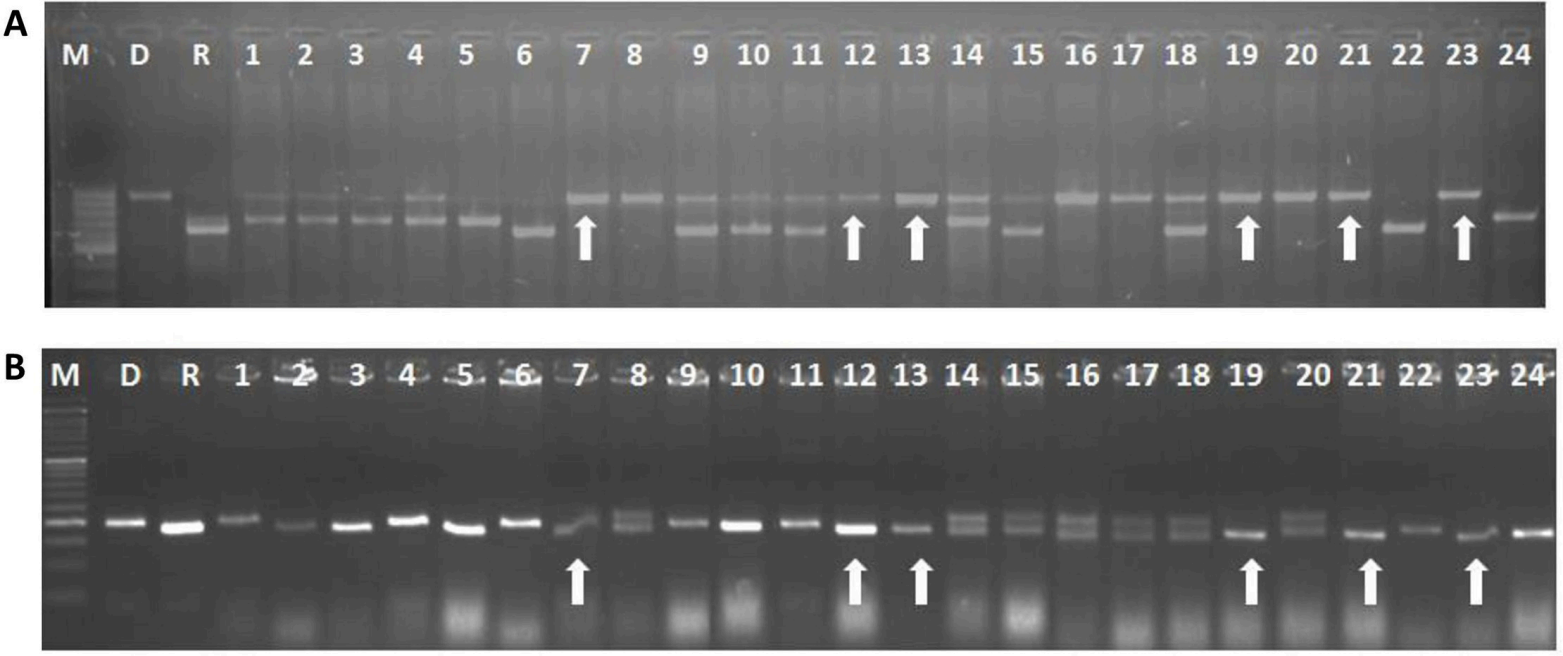
Screening of ICF_2_ population for identification of double homozygotes for the target resistancegenes, viz., *Xa21* and *Xa33*. The ICF_2_ plants were screened through PCR to analyze the allelic status of *Xa21* (A) and *Xa33* (B) using the gene-specific markers. M–Marker, R–Recurrent parent (i.e. DRR17B) and D–donor parent [i.e., ‘**ISM**’ (A) and FBR1-15EM (B)]. Arrows indicate plants which possess target genes *Xa21* and *Xa33* in homozygous condition.

**Fig 3:**
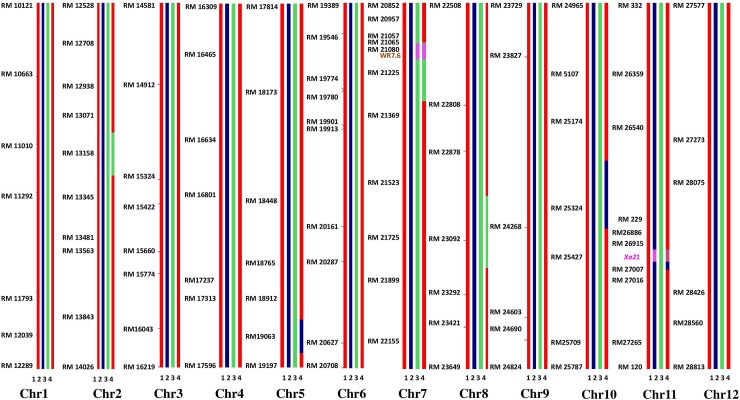
Graphical genotyping representation for the best improved line (IIRRIC102-26-7) of DRR17B. Graphical genotyping representing that the best line of improved two-gene-containing DRR17B line (*Xa21*+ *Xa33*), IIRRIC102-26-7 exhibiting the highest genome recovery of the recurrent parent with more than 95%, along with minimum linkage drag on carrier chromosomes 7 and 11, with less than 2 Mb donor parent chromosome (1. DRR17B, 2. ‘**ISM**’, 3. FBR1-15, 4. IIRRIC102-26-7).

The true F_1_s derived by crossing DRR17B with ‘**ISM**’ (i.e., Cross I) and FBR1-15 (i.e., Cross II) were backcrossed with DRR17B to obtain BC_1_F_1_s, which were then screened with the gene-specific markers. A total of 61 and 65 BC_1_F_1_ plants were observed to be positive for the target genes in Cross I and Cross II, respectively. The positive plants were screened with markers specific for *Rf3* and *Rf4*,and a total of 15 and 11 plants were identified to be devoid of both the fertility restorer genes concerning Cross I and Cross II, respectively. These plants were then subjected to background selection using a set of polymorphic SSR markers (61 markers for Cross I and 64 for Cross II). Plant # IIRRGP3 from Cross I, with a recurrent parent genome (RPG) recovery of 73.7% and Plant # IIRRGP22 from Cross II, with a RPG recovery of 75% were identified to be the best ones (i.e. having a maximum recovery of DRR17B genome) and were used for further backcrossing. The process of marker-assisted backcrossing was carried out until BC_3_F_1_ generation (details given in **Table 2**). At BC_3_F_1_, plant # IIRRGP3-87-64 from Cross I with RPG recovery of 93.4% and plant # IIRGP22-73-10 with RPG recovery of 93.7% were identified to be superior and were selfed to generate BC_3_F_2_s. With regards to the BC_3_F_2_s produced from Cross I and Cross II, 39 and 52 plants were identified to be homozygous for *Xa21* and *Xa33*, respectively. Among these, a solitary plant, which was morphologically similar to DRR17B, was identified from Cross I (i.e., plant # IIRRGP 3-87-64-22 and Cross II (i.e., plant # IIRRGP 22-73-10-15) and intercrossed with each other to generate intercross F_1_s (i.e., ICF_1_s). Out of 68 ICF_1_s, 63 were identified to be heterozygous for both *Xa21* and *Xa33* (i.e. true intercross F_1_s), and they were selfed to obtain ICF_2_ generation. At ICF_2_, a total of 309 plants were screened with markers specific for *Xa21* and *Xa33* and 18 were identified to be double homozygotes (**Table 2**; **Fig 2**). A total of nine plants out of the 18, which were identified to be phenotypically similar to DRR17B, were further advanced until ICF_4_ generation through phenotype-based pedigree selection. At ICF_4_ generation, six promising lines which were similar to the recurrent parent were identified (line #IIRRIC 10-8-94, IIRRIC 10-19-138, IIRRIC 102-26-7, IIRRIC 123-34-84, IIRRIC 123-58-3 and IIRRIC 172-77-12) and analysed for their resistance to BB, sterility maintenance ability and also characterized for important agro-morphological traits. Among the six ILs, line # IIRRIC102-26-7 exhibited the highest recurrent parent genome recovery with more than 95% along with minimal linkage drag on carrier chromosomes (**Fig 3**).

**Table 2:** Details of plants generated and analyzed with markers in each generation of backcrossing/intercrossing.

**S. No.**	**Generation**	**No. of plants screened**		**No. of positive plants for target genes and negative *Rf3* and *Rf4***		**Recurrent parent genome recovery (%) of the selected backcross plant**	
		***Xa21***	***Xa33***	***Xa21***	***Xa33***	***Xa21***	***Xa33***
1	BC_1_F_1_	187	134	15	11	73.7	75.0
2	BC_2_F_1_	112	157	42	59	85.2	85.9
3	BC_3_F_1_	144	142	47	48	93.4	93.75
4	BC_3_F_2_	178	213	39	52	-	-
		**No. of intercross plants screened**		**No. of homozygous double positive plants for *Xa21* and *Xa33***			
5	ICF_1_	68		63		—	
6	ICF_2_	309		18		—	

### Phenotypic evaluation of ILs for BB resistance

**Fig 4:**
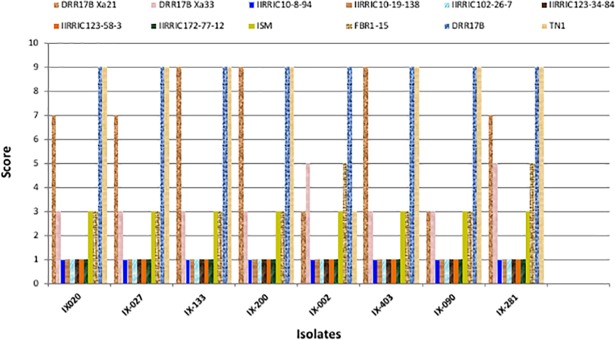
Screening of the single-gene and two-gene pyramid lines of DRR17B against different virulent isolates of the bacterial blight pathogen. Eight selected ICF_4_of DRR17B possessing either *Xa21 (*IIRRGP3-87-64-22-4-50) or *Xa33* (IIRRGP22-73-10-15-13-2) or Xa21 + *Xa33* (# IIRRIC10-8-94, IIRRIC10-19-138, IIRRIC102-26-7, IIRRIC123-34-84, IIRRIC123-58-3, and IIRRIC172-77-12) were screened for their BB resistance with eight virulent isolates of *Xanthomonas oryzae* pv. *Oryzae* (*Xoo*) along with the recurrent parent (DRR17B) and donor parents (‘**ISM**’ and FBR1-15). While all the lines showed excellent resistance against the multiple isolates of *Xoo* screened, the two-gene pyramid lines (i.e., *Xa21 + Xa33*) were observed to show a higher level of resistance to the different isolates of the pathogen.

The recurrent parent, DRR17B (11) (with lesion lengths ranging from 18.8 to 33.1 cm) and susceptible check TN1 (12) (with lesion lengths ranging from 20.9 to 33.8 cm) showed a disease score of 9 against all the eight isolates of the *Xoo* (**Table 3**; **Fig 4** depicted as graph). The resistant check and the donor for *Xa21* gene ‘**ISM**’ (9) (possessing *Xa21*, *xa13*, and *xa5*) showed a score of 3 against all the isolates (with an average lesion length ranging from 1.6 to 3.6 cm). FBR1-15 (10), the donor for *Xa33* gene and improved DRR17B lines possessing *Xa33* (# IIRRGP22-73-10-15-13-2 (2)) showed a resistance score of 3 with most of the isolates (with lesion lengths ranging from 1.7 to 4.8 cm), with two isolates, IX-002 and IX-281 recorded moderate resistance reaction with a score of 5 (average lesion lengths ranging from 7.3 to 9.7 cm and 7.5 to 9.2 cm).The ILs of DRR17B containing only *Xa21* (# IIRRGP3-87-64-22-4-50 (1)) showed a resistance reaction against two isolates *viz*., IX-002 and IX-090 with a score of 3 (with lesion lengths of 2.8 to 4.3 cm and 2.0 to 2.8 cm, respectively), while with three isolates,*viz*., IX-020, IX-027 and IX-281, the line with only *Xa21* exhibited moderately susceptibility with a score of 7 (with lesion lengths of 12.5 to 14.7 cm, 13.1 to 14.5 cm and 13.0 to 14.6 cm, respectively). Further, the line showed highly susceptible reaction with a score of 9 (with lesion lengths of 20.1 to 23.5cm, 22.2 to 25.6 cm and 21.9 to 24.4cm, respectively)with three other three isolates *viz*., IX-133, IX-200 and IX-409, respectively. The ILs of DRR17B containing both *Xa21* + *Xa33* (3–8) exhibited a significantly higher level of resistance, showing a score of 1 against all eight isolates with lesion lengths ranging from 0.1 to 1 cm (**Table 3**; **Fig 4**).

**Table 3:** Reaction of the breeding lines of DRR17B possessing *Xa21* and *Xa33*, singly or in combination against eight virulent isolates of the bacterial blight pathogen, *Xanthomonas oryzae* pv. *oryzae (Xoo)*.

*Xoo* Isolates		Breeding Lines								Parents/Resistant/Susceptible Check				CV	LSD	H^2^	F Value
		1	2	3	4	5	6	7	8	9	10	11	12				
**IX-020**	Mean ± SE	13.66 ± 0.42 b	3.8 ± 0.28c	0.68 ± 0.07e	0.5 ± 0.06e	0.58 ± 0.06e	0.66 ± 0.07e	0.54 ± 0.05e	0.56 ± 0.05e	2.66 ± 0.19d	3.68 ± 0.27cd	31.02 ± 0.87a	30.12 ± 0.95a	11.5	1.08	0.99	909.86
	Range	12.5–14.7	3.2–4.7	0.5–0.9	0.3–0.7	0.4–0.7	0.5–0.7	0.4–0.7	0.5–0.7	2.1–3.1	3.0–4.4	28.6–33.1	27.6–32.8				
	Score	7	3	1	1	1	1	1	1	3	3	9	9				
**IX-027**	Mean ± SE	13.88 ± 0.24c	4.08 ± 0.24d	0.28 ± 0.10e	0.44 ± 0.07e	0.42 ± 0.07e	0.34 ± 0.06e	0.30± 0.09e	0.36 ± 0.09e	2.90 ± 0.23d	3.94 ± 0.30d	21.60 ± 0.90b	25.20 ± 1.30a	17.8	1.39	0.99	335.00
	Range	13.1–14.5	3.5–4.9	0.1–0.6	0.2–0.6	0.2–0.6	0.2–0.5	0.1–0.6	0.1–0.6	2.1–3.5	3.1–4.8	18.8–23.5	20.9–28.5				
	Score	7	3	1	1	1	1	1	1	3	3	9	9				
**IX-133**	Mean ± SE	22.04 ± 0.79c	3.56 ± 0.25d	0.2 ± 0.00f	0.24 ± 0.02f	0.26 ± 0.04f	0.26± 0.04f	0.28 ± 0.04f	0.22 ± 0.02f	2.2 ± 0.19e	3.4 ± 0.21d	24.14 ± 0.75b	26.06 ± 0.57a	11.91	1.04	0.99	809.23
	Range	20.1–23.5	2.7–4.1	0.2	0.2–0.3	0.2–0.4	0.2–0.4	0.2–0.4	0.2–0.3	1.7–2.7	2.8–3.9	22.9–26.5	24.8–27.5				
	Score	9	3	1	1	1	1	1	1	3	3	9	9				
**IX-200**	Mean ± SE	23.42 ±0.70b	3.52 ± 0.21c	0.82 ± 0.08d	0.78 ± 0.04d	0.7± 0.07d	0.76 ±0.08d	0.68 ± 0.08d	0.64 ±0.07d	2.74 ± 0.18c	3.68 ± 0.17c	30.56 ± 0.76a	31.62 ± 0.79a	10.49	1.11	0.99	1006.00
	Range	22.2–25.6	3.0–4.2	0.6–1	0.7–0.9	0.5–0.9	0.5–1	0.4–0.8	0.4–0.8	2.3–3.1	3.3–4.2	28.2–32.8	29.3–33.8				
	Score	9	3	1	1	1	1	1	1	3	3	9	9				
**IX-002**	Mean ± SE	3.72 ± 0.26c	8.58 ± 0.49b	0.86 ± 0.05e	0.74 ± 0.07e	0.8 ±0.04e	0.72 ± 0.06e	0.84 ± 0.05e	0.8 ± 0.04e	2.34 ± 0.22d	8.72 ± 0.43b	24.88 ± 0.90a	24.76 ± 0.65a	13.22	1.09	0.99	558.46
	Range	2.8–4.3	7.2–9.8	0.7–1	0.5–0.9	0.7–0.9	0.6–0.9	0.7–1	0.7–0.9	1.8–2.7	7.3–9.7	22.6–27.7	22.5–26.2				
	Score	3	5	1	1	1	1	1	1	3	5	9	3				
**IX-403**	Mean ± SE	23.34 ± 0.53b	2.52 ±0.15c	0.48 ± 0.06d	0.52 ±0.07d	0.48 ± 0.06d	0.5 ± 0.07d	0.48 ±0.06d	0.52 ± 0.02d	2.28 ± 0.19c	2.84 ± 0.17c	21.34 ± 0.83a	25.92± 0.98a	13.21	1.19	0.99	658.00
	Range	21.9–24.4	2–2.7	0.3–0.6	0.3–0.7	0.4–0.7	0.3–0.7	0.3–0.6	0.5–0.6	1.6–2.7	2.4–3.3	23.9–28.1	23.4–28.8				
	Score	9	3	1	1	1	1	1	1	3	3	9	9				
**IX-090**	Mean ± SE	2.46 ± 0.13cd	2.88 ±0.17c	0.56 ± 0.04e	0.56 ± 0.06e	0.54 ± 0.07e	0.58 ± 0.06e	0.54 ±0.04e	0.6 ± 0.04e	1.96 ± 0.26d	3.22 ± 0.19c	25.78 ± 0.74b	29.42 ± 0.66a	12.03	0.88	0.99	1101.20
	Range	2–2.8	2.4–3.5	0.5–0.7	0.4–0.7	0.3–0.7	0.4–0.7	0.5–0.7	0.5–0.7	1.7–2.8	1.7–3.7	20.9–28.5	23.2–31.6				
	Score	3	3	1	1	1	1	1	1	3	3	9	9				
**IX-281**	Mean ± SE	13.68 ± 0.40c	8.3 ± 0.45d	0.54 ± 0.09f	0.56 ± 0.04f	0.62 ± 0.08f	0.66 ± 0.05f	0.46 ± 0.05f	0.6 ± 0.04f	2.92 ± 0.22e	8.24 ±0.30d	24.1 ± 0.56b	28.68 ± 0.55a	9.43	1.53	0.99	987.94
	Range	13.0–14.6	7.1–9.4	0.3–0.8	0.5–0.7	0.4–0.8	0.5–0.8	0.3–0.6	0.5–0.7	2.4–3.6	7.5–9.2	23.5–25.7	27.9–30.3				
	Score	7	5	1	1	1	1	1	1	3	5	9	9				

Breeding line **1** represents DRR17B line containing *Xa21* gene that was screened with eight different isolates under glass house conditions at IIRR. With two isolates (*viz*., FZB and Lud-05-1 *Xa21*) introgressed lines that showed resistance reaction having a score of 3. With remaining isolates, *Xa21* introgressed lines showed moderate susceptibility with ascore of 7 and high susceptibility having a score of 9 (Lore et al., 2011). Breeding line **2** represents DRR17B line containing *Xa33* gene, except for two isolates (*viz*., FZB and TNK12-3 moderate resistance with a score of 5) while the remaining isolates showed a resistance reaction score of 3. Breeding lines **3–8** represent ILs of DRR17B containing *Xa21* + *Xa33* genes that exhibited a high level of resistance with a score of 1 against all eight isolates. Resistant checks **9** and **10** represent ‘**ISM**’, which showed resistance against all eight isolates with a score of 3 and another resistant check, FBR1-15, which showed resistance reaction score of 3 except the two isolates that showed moderate resistance with a score of 5. Recurrent parents **11** and **12** represent DRR17B and susceptible check TN1 that exhibited highly susceptible reactions against all eight isolates with a score of 9. (**1-**IIRRGP3-87-64-22-4-50 (*Xa21*), **2-**IIRRGP22-73-10-15-13-2 (*Xa33*), **3-**IIRRIC10-8-94, **4-**IIRRIC10-19-138, **5-**IIRRIC102-26-7, **6-**IIRRIC123-34-84, **7-**IIRRIC123-58-3,**8-**IIRRIC172-77-12, **9-‘ISM**’, **10-**FBR1-15, **11-**DRR17B and **12-**TN1).

### Characterization of ILs for maintenance ability and agro-morphological traits

**Fig 5:**
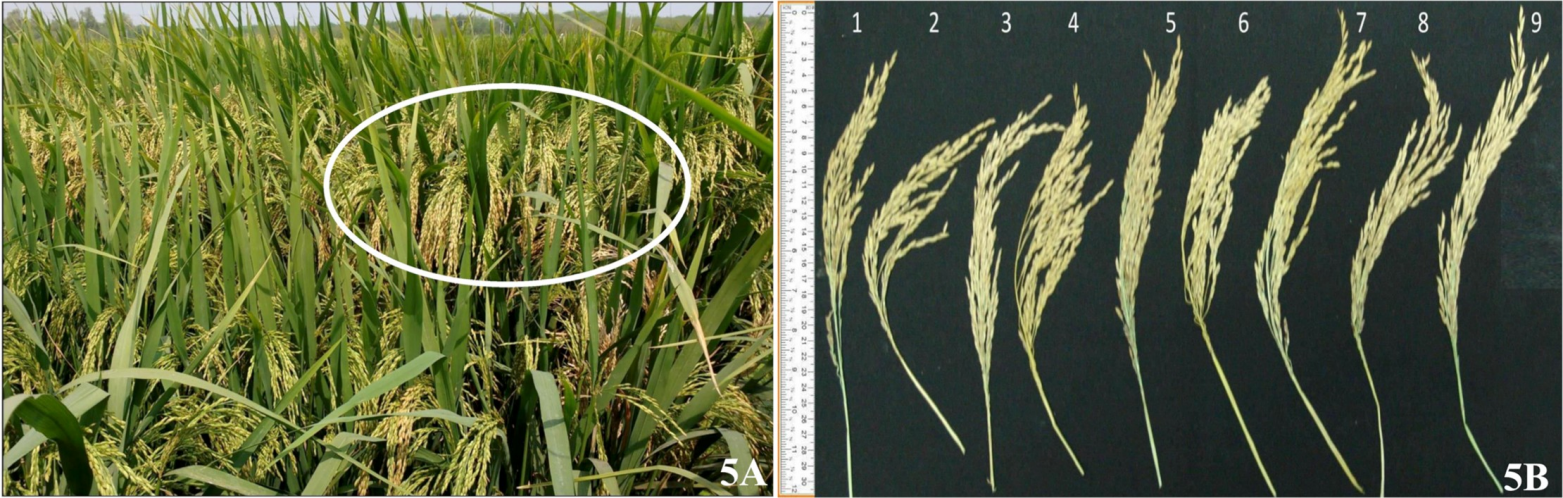
IL of DRR17B displaying high grain number under field conditions at IIRR, Panicles of ILs along with donor and recurrent parents. **A.** IL # IRIC102-26-27 displaying high grain number in field conditions, **B.** The ILs of DRR17B possessing *Xa21* + Xa33 genes(# IIRRIC10-8-94, IIRRIC10-19-138, IIRRIC102-26-7, IIRRIC123-34-84, IIRRIC123-58-3, and IIRRIC172-77-12)were displayed more number of grains per panicles and similar panicle length (except number 6) when compared with DRR17B.

The current study screened the six ILs for their maintenance ability. Out of six, three lines showed partial spikelet fertility, while the remaining three lines (viz., line # IIRRIC102-26-7, IIRRIC123-34-84, and IIRRIC172-77-12) showed complete spikelet sterility when crossed with the WA-CMS line, IR58025A (**Table 4**). Comparison of five agro-morphological parameters (days to 50% flowering, plant height, number of productive tillers, panicle length and number of grains per panicle) revealed thatall the six ILs are isophenic in their panicle length and number of productive tillers to DRR17B, while significant differences were observed with respect to the number of grains per panicle. The ILs viz., IIRRIC10-8-94, IIRRIC102-26-7, IIRRIC123-58-3 and IIRRIC172-77-12 possessed more number of grains per panicle with respective to DRR17B viz., 301.6, 360.4, 308 and 317 respectively (**Fig 5A and 5B**). However, all selected six lines showed comparatively shorter plant height than recurrent parent. While panicle length of, line # IIRRIC102-26-7 was observed longest among all six panicle (24.16 cm), the remaining five ILs exhibited equal or less than the recurrent parent DRR17B (average length of 23.24 cm: **Table 4**). Line # IIRRIC102-26-7 exhibit highest numbers of productive tillers per plant (average of 12), all remaining five ILs were similar to thir recurrent parent (10–11.2). The to 50% flowering, of all the six ILs flowered earlier (92–102 days), as compared to DRR17B (105 days).

**Table 4:** Agro-morphological features of selected backcross derived lines of DRR17B possessing *Xa21 + Xa33*.

Plant identity	Days to 50% flowering	Plant height in cm		No. of productive tillers		Panicle length in cm		No. of grains per panicle		Spikelet fertility (%) (IR58025A X selected ICF4 Plant)
		Mean ± SE	Range	Mean ± SE	Range	Mean ± SE	Range	Mean ± SE	Range	
**IIRRIC10-8-94**	97	81.84 ± 1.51de	76.8–86.8	10.6 ± 1.03a	8–14	23.64 ± 0.60a	21.9–25.5	301.6 ± 7.14bc	284–322	2
**IIRRIC10-19-138**	92	78.36 ± 1.19ef	74.1–82.4	10 ± 0.71a	8–12	21.52 ± 1.05b	18.7–23.8	263.8 ± 9.49de	235–294	5
**IIRRIC102-26-7**	101	89.86 ± 1.18b	85.1–93.2	12 ± 1.10a	10–16	24.16 ± 0.63a	21.9–25.5	360.4 ± 9.98 a	331–390	0
**IIRRIC123-34-84**	95	78.62 ± 1.10ef	74.7–82.4	10.8 ± 1.16a	8–14	23.28 ± 0.45ab	21.7–24	279.8 ± 8.86cd	265–311	0
**IIRRIC123-58-3**	95	84.92 ± 1.21cd	80.6–89.4	11.2 ± 1.11a	7–13	22.52 ± 0.65ab	20–23.1	308.0 ± 9.48b	278–328	18
**IIRRIC172-77-12**	97	77.06 ± 1.13f	81.1–73.2	11.2 ± 1.20a	9–15	21.62 ± 0.72b	19.2–23.4	317.0 ± 7.00b	298–339	0
**DRR17B**	105	96.08 ± 1.86a	90.6–102.1	10.8 ± 0.97a	9–14	23.24 ± 0.64ab	21.7–25.6	280.0 ± 11.60cd	284–307	-
**ISM**	108	86.78 ± 1.04bc	83.1–90.3	11.6 ± 1.21a	9–15	19.58 ± 0.72c	17.7–21.9	252.0 ± 7.98e	224–270	-
**FBR1-15**	103	77.02 ± 1.34f	73–82.1	9.8 ± 0.66a	8–12	18.76 ± 0.72c	16.0–19.9	200.2 ± 11.10f	169–228	-
**CV**	-	4.22	-	22.11	-	6.6	-	7.02	-	-
**LSD (p = 0.05)**	-	4.5287	-	3.1018	-	1.8724	-	25.734	-	-
**H**^**2**^	-	0.88	-	0.25	-	0.68	-	0.9	-	-
**F**	-	17.6	-	0.43	-	8.14	-	25.52	-	-

Values in a column followed by common letters do not differ significantly by Duncan’s Multiple Range Test (DMRT). DMRT (P = 0.05)

## Discussion

Several studies indicate that global rice production needs to be doubled by 2050 to meet the demands of ever growing population [2]. However, rice grain yield is badly affected by biotic and abiotic stresses [31]. The present study was taken up to improve, an elite maintainer of rice, DRR17B, for its resistance against BB resistance. DRR17B is a fine grain type and medium duration maintainer line of rice, possessing stable maintenance ability was developed by ICAR-Indian Institute of Rice Research, Hyderabad, India [23]. As DRR17A and its maintainer parent- DRR17B are highly susceptible to BB disease, considering this deficiency in the elite maintainer line, the current study was carried out with an objective to introgress two major dominant BB resistant genes, *viz*., *Xa21* and *Xa33* through MABB in order the make the maintainer line durably resistant to BB. These two selected genes are known to confer resistance against multiple isolates of the BB pathogens for large rice cultivated area; hence, the hybrids developed from ILs of DRR17A will also be sustainable resistant against this disease.

Introgression of BB resistance genes through conventional breeding involving patho-phenotypic selection which is very laborious, time and resource consuming process and its success significantly depends on accurate disease scoring, the existence of environmental conditions which favour disease development and the availability of appropriate virulent strains of the pathogen causing the disease [11]. As compared to conventional breeding, marker-assisted selection (MAS) breeding strategy is more useful for targeted introgression of resistance genesas it does not depend on the availability of virulent strains or existence of ideal environmental conditions, since the selections are indirect, and are based on the presence or absence of specific alleles of molecular markers linked to the resistance genes. Previous studies, [23, 32, 33] reported on successfully developed bacterial blight resistant versions of hybrid rice parental lines PRR78 and IR58025B, through marker-assisted selection for target traits in the initial stages and phenotype-based selection at later stages and hence at the same methodology was adopted in the current study.

So far, at least 41 genes conferring resistance against BB have been identified in rice [11, 12, 13]. Among them, the wild rice derived gene, *Xa21* encoding a receptor kinase-like protein has been successfully deployed by many research groups across the world, as it has been documented to confer broad-spectrum resistance against the BB disease [17, 18, 23, 32, 34, 35, 36, 37, 38]. The commonly used BB resistance gene *Xa21* has been tagged and mapped on chromosome 11 with a tightly-linked PCR-based marker pTA248 [21].*Xa33*, the wild rice derived BB resistance gene has been reported to confer broad spectrum resistance [22] and the gene has been deployed by the research group at Tamil Nadu Agricultural University, Coimbatore, India and the breeding lines possessing *Xa33* were observed to be very effective in terms of their BB resistance [39, 40]. Hence, these two broad spectrum resistance genes were selected for introgression into the DRR17B.

Phenotypic screening for BB resistance was carried out in this study among selected single gene containing BC_3_F_6_ lines possessing either *Xa21* or *Xa33* and two-gene containing intercross derived lines at ICF_4_ generation possessing *Xa21*+*Xa33* along with the donor and recurrent parents (‘**ISM**’, FBR1-15, and DRR17B, respectively) using eight virulent isolates of *Xoo*. All the ILs possessing *Xa21+Xa33* were observed to show significantly higher level of resistance against BB when compared to the donor parents, ‘**ISM**’ and FBR1-15. Single gene containing lines of DRR17B (i.e. possessing either *Xa21* or *Xa33*), the recurrent parent DRR17B and the BB susceptible check TN1 (**Table 3; Fig 4**).It is earlier known that *Xa21* confers broad spectrum resistance against many of the virulent pathotypes of *Xoo* in India [17,18] and several studies have indicated the suitability of *Xa21* in BB resistance gene pyramiding programmes [10, 18, 34, 41, 42]. However, in this study, a few isolates of the pathogen were observed to be compatible with *Xa21* containing lines of DRR17B indicating that *Xoo* isolates, which are capable of overcoming *Xa21* conferred resistance are fast-developing [17, 43, 44]. Interestingly, the ILs of DRR17B possessing *Xa33*were observed to show a better level of resistance as compared to the lines having *Xa21*. Furthermore, DRR17B lines possessing both *Xa21* and *Xa33* were observed to be highly resistant against all the eight virulent isolates of *Xoo*, thus, indicating the suitability of deployment of *Xa33* either singly or in combination with *Xa21*. Earlier, two elite restorer lines, KMR3R, and RPHR1005 were improved for BB resistance by introducing *Xa21* [23, 33, 36, 38]. Similarly, *Xa33* has been deployed in different genetic backgrounds by different research groups [19, 22, 39, 40]. However, this is the first report wherein *Xa21* has been combined with *Xa33* in the genetic background of an elite maintainer line, i.e., DRR17B and the gene-pyramid lines demonstrated a higher level of resistance as compared to lines possessing a single resistance gene (**Table 3; Fig 4**).

It is a known fact that long term cultivation of rice varieties possessing single resistance gene can result in the breakdown of resistance by faster development of virulent pathogens [43, 44, 45].Hence, pyramiding of multiple resistance genes has been advocated to be one of the best strategies to achieve durable dual-resistance [18, 46, 47]. In our present study, the genotype ‘**ISM**’ (with *Xa21* + *xa13* + *xa5*) has displayed satisfactory level of resistance with a score of 3 against all eight isolates. Interestingly, when *Xa21* gene was combined with another major dominant gene- *Xa33*, such breeding lines exhibited the highest level of resistance with a score of 1. This indicates that the gene combination *Xa21 + Xa33* displayed a broad spectrum of resistance and hence can be recommended for deployment in hybrid rice improvement programs as both *Xa21* and *Xa33* are both dominant and will express in the F_1_ hybrid.

Similar to the approach adopted in the current study, several earlier studies also resorted to phenotype-based selection for key agro-morphological traits along with marker-assisted selection while improving elite varieties and parental lines for one or more target traits through MABB [18, 23, 33, 35, 36, 37, 38, 48].The approach of deployment of MABB strategy for the target resistance genes along with negative selection for major fertility restorer genes, *Rf3* and *Rf4*, coupled with phenotype-based selection for certain key agronomic characters helped in near-complete recovery of good features of DRR17B along with identification of few ILs with complete maintenance ability (**Table 4**). In addition to improving BB resistance of DRR17B, The current study also focused on the identification of ILs of DRR17B possessing plant height which is significantly shorter than DRR17B, as shorter plant is preferred for deployment as good maintainers [23]. Significant differences in plant height were observed in many improved DRR17B lines *viz*., RMSIC 10-8-94, RMSIC 10-19-138, RMSIC 102-26-7, RMSIC 123-34-84, RMSIC 123-58-3 and RMSIC 172-77-12 and these lines could serve as better maintainers as compared to DRR17B. Interestingly, when compared to DRR17B, some of the ILs exhibited advantage concerning grain number per panicle. These lines include RMSIC 10-8-94, RMSIC 102-26-7, RMSIC 123-58-3 and RMSIC 172-77-12 (**Fig 5A** and **5B**).For the panicle length, all the ILs showed values equivalent to DRR17B, except one line *viz*., RMSIC 102-26-7, a wherein slight improvement over the recurrent parent was noticed. Significant differences (i.e., shorter duration) were observed concerning number of days to 50% flowering in some of the backcross derived plants (**Table 4**). No significant differences were observed between improved versions of DRR17B and recurrent parent DRR17B concerning other agro-morphological characters or grain type and the lines mostly resembled the original recurrent parent. The approach of coupling of MABB with phenotypic selection adopted in this study helped to regain most of the key agro-morphological traits of DRR17B, while simultaneously helping in the selection of some superior backcross derived segregants of DRR17B possessing BB resistance.

The ILs of DRR17B were test crossed with IR58025A (WA-CMS line) to check their maintainer ability. Three lines (*viz*., IIRRIC102-26-7, IIRRIC123-34-84, and IIRRIC172-77-12) displayed complete maintainer ability highlighting the necessity of phenotypic confirmation for maintenance ability, despite a rigorous marker-assisted selection for *rf3* and *rf4* alleles in this study. This could be attributed to the existence of minor fertility restorer genes/QTLs as reported earlier [49].The three ILs of DRR17B, possessing *Xa21 + Xa33* are being converted as CMS lines by crossing with DRR17A through MABB.

The six ILs of DRR17B exhibited high level of BB resistance against the BB isolates, when compared with the recurrent parent DRR17B. Whereas in agro-morphological characters like plant height, day to 50% flowering and number of grains per panicle etc, variations were observed. All the improved lines were shorter than the recurrent parent. With regards to Days to 50% flowering all the improved lines were little early (92–103 days) than DRR17B (105 days). Some the improved lines *Viz*., IIRRIC10-8-94, IIRRIC102-26-7 IIRRIC123-58-3 and IIRRIC172-77-12 were exhibited significantly more number of grains per panicle then DRR17B (280 per panicle). The ILs Viz., IIRRIC102-26-7, IIRRIC123-34-84, and IIRRIC172-77-12 were exhibited complete maintainer ability as like DRR17B and remaining lines were partial maintainers.

## Conclusion

The present study has resulted in development of improved versions of an elite maintainer of rice, DRR17B possessing durable resistance against BB through incorporation of two major dominant genes conferring broad-spectrum resistance, *Xa21* and *Xa33* by marker-assisted backcross breeding (MABB) strategy. The double gene pyramided lines of DRR17B expressed high level of resistance against eight different virulent isolates of *Xoo* and their resistance levels was comparable with triple resistance gene pyramided rice variety, ‘**ISM**’ (possessing *Xa21* + *xa13* + *xa5*) and were also significantly better than the single gene containing lines (possessing *Xa21* or *Xa33*). Three promising double-gene pyramided lines of DRR17B with high level of BB resistance, agro-morphological attributes similar to or superior to the DRR17B with complete maintainer ability would be helpful in development of superior rice hybrids with durable, broad-spectrum resistance.
